# Tardive Oropharyngeal Dyskinesia Associated With Antipsychotic Use in the Management of Behavioral and Psychological Symptoms of Dementia

**DOI:** 10.14740/jmc5277

**Published:** 2026-04-29

**Authors:** Zahira Zohari, Nurulakmal Obet, Nurul Huda Mohd Zambri, Reena Nadarajah, Dato’ Tunku Muzafar Shah Tunku Jaafar

**Affiliations:** a Geriatric Unit, Medical Department, Faculty of Medicine and Health Sciences, Universiti Putra Malaysia, Selangor, Malaysia; b Geriatric Unit, Medical Department, Hospital Kuala Lumpur, Malaysia; c Geriatric Unit, Medical Department, Hospital Selayang, Selangor, Malaysia

**Keywords:** Dementia, Antipsychotics, Psychiatry, Tardive dyskinesia, Extrapyramidal symptoms

## Abstract

Behavioral and psychological symptoms of dementia (BPSD) are prevalent in up to 90% of people with dementia. Management of BPSD often includes non-pharmacological approaches, supplemented by psychotropic medications when necessary. However, these medications can lead to adverse reactions, including extrapyramidal symptoms (EPS). This report describes the case of an older adult presenting with BPSD secondary to vascular dementia. The management of the BPSD included various psychotropic and antipsychotic medications, which led to the development of orofacial dyskinesias. The development and recurrence of tardive dyskinesia in this patient were explored, considering the delayed effects of typical antipsychotics, the potentiating effects of atypical antipsychotics, and interactions with selective serotonin uptake inhibitors and acetylcholinesterase inhibitors. The case highlights the complexities of managing BPSD in dementia and the risk of EPS. It emphasizes the importance of early recognition and the value of teleconsultation for close monitoring and medication review, thereby improving patient well-being and treatment adherence. An evidence-informed approach, combined with regular medication review and early identification of adverse effects, is essential to optimize therapeutic outcomes and mitigate long-term morbidity associated with the pharmacological management of BPSD.

## Introduction

Behavioral and psychological symptoms of dementia (BPSD) occur in up to 90% of people with dementia (PWD) [1, 2]. BPSD is associated with high levels of distress both in people living with dementia and their caregivers, adverse outcomes, and increased use of health care resources. Management of BPSD includes non-pharmacological approaches such as cognitive stimulation, physical activity, music therapy, phototherapy, and aromatherapy. When these are insufficient, the use of psychotropic medications and antipsychotics becomes necessary.

The usage of these medications is not without consequences. Among the known side effects is the development of extrapyramidal symptoms (EPS). EPS refers to unusual movements resulting from the blockade of the dopamine pathway, typically iatrogenic [3]. It is associated with the blockade of post-synaptic dopamine (D2) receptors, which can lead to increased dopamine receptor sensitivity. EPS includes acute dyskinesia and dystonia, tardive dyskinesia (TD), Parkinsonism, akinesia, akathisia, and neuroleptic malignant syndrome. These symptoms significantly impact a patient’s quality of life. The involuntary movements can range from mild to severe, socially stigmatizing and emotionally distressing for those affected.

We present a complex and challenging case of BPSD secondary to vascular dementia with complications during pharmacological management of his symptoms. There appear to be few reported cases regarding the essence of this problem and its management.

This case report aims to highlight the risk of EPS, particularly TD, in the pharmacological management of BPSD, and to emphasize the importance of careful prescribing and close monitoring in older adults.

## Case Report

### Initial diagnosis

We report a case of a 78-year-old man, a food stall operator, with a medical history of hypertension, dyslipidemia, and a right-sided stroke in January 2021 with minimal residual weakness. He was independent of basic activities of daily living (ADLs) at presentation.

In February 2021, he was brought to the psychiatric clinic by his primary carer, a long-time helper at his noodle stall, who noted behavioral changes and cognitive decline a month after the stroke. He exhibited irritability, unprovoked outbursts, visual hallucinations, and disrupted sleep, leading to daytime agitation. His Folstein mini-mental state examination (MMSE) score on the initial review was 19/30 [4]. He was diagnosed with major neurocognitive disorder with BPSD.

At that point, the psychiatry team initiated haloperidol, a typical antipsychotic, at twice daily dosing at 0.75 mg in the morning and 1.5 mg at night, which led to improvement. After 10 months, the frequency was reduced to 1.5 mg once nightly (January 2022).

He was referred to our Geriatric Clinic for further management in February 2022. A comprehensive geriatric assessment confirmed a cognitive decline. The patient demonstrated a decline in instrumental ADLs and cognitive responsiveness. His behavior and visual hallucinations had improved. He was given a diagnosis of mild vascular dementia with BPSD. The haloperidol was reduced to a PRN basis only.

### Development of TD

Subsequent evaluation in May 2022 revealed sleep difficulties, agitation, and increased visual hallucinations. On this occasion, risperidone, an atypical antipsychotic, was introduced at 0.5 mg at night. This resulted in improved sleep and cessation of the hallucination. Given the prominence of the behavioral symptoms, clinical attention was directed towards managing BPSD, and the use of cognitive enhancers was deferred.

After approximately 6 weeks on risperidone, daytime somnolence became an issue. During teleconsultation, the medication was withheld. Upon review, 5 days after the teleconsultation, he demonstrated abnormal tongue, lip, and head movement, which were clearly apparent. There was no rigidity or tremor in the upper limbs. The rest of the physical examination and blood investigations were unremarkable. There was no prior history of movement disorders or neuroleptic exposure before 2021.

There was a concern of EPS, TD secondary to risperidone. Thus, the decision was made to discontinue the antipsychotics altogether. Donepezil, an acetylcholinesterase inhibitor, 5 mg daily, was commenced to address his cognitive decline. Teleconsultation after 2 weeks revealed complete cessation of the abnormal movement. He remained off antipsychotics.

Unfortunately, in October 2022, there was a recurrence of vivid hallucinations, nocturnal agitation, and wandering, significantly impacting the patient’s and carer’s quality of life. Haloperidol 0.75 mg at night was reintroduced in view of previous efficacy. We also added escitalopram 5 mg at night, a selective serotonin uptake inhibitor (SSRI), to address the agitation.

By March 2023, there was a further cognitive decline characterized by increased disorientation, although he remained independent in his basic ADLs. He appeared quiet and disinterested, exhibiting unsteadiness with several near falls. Correspondingly, the agitation, wandering, and hallucination had subsided. Clinically, he appeared sedated, and the abnormal movements reappeared, consisting of tongue protrusion, drooling, and involuntary lip movements. No head nodding or abnormal neck movements were observed. There were no features suggestive of parkinsonism. Haloperidol was discontinued as a result. The escitalopram was maintained, and the donepezil was increased to 10 mg daily.

Unfortunately, in April 2023, just 2 weeks later, he sustained a closed right intertrochanteric fracture after a fall, requiring surgical intervention. While an inpatient, he was reported to have disrupted sleep and visual hallucinations with abnormal movements, consisting of head nodding and orolingual dyskinesia. No pharmacological changes were made, and he remained on donepezil and escitalopram at this point as the symptoms were attributed to delirium, and management focused on non-pharmacological interventions.

In May 2023, the patient revealed clinical improvement. His dyskinesia improved with minimal head nodding, and his mood notably better, with reduced aggression, irritability, and visual hallucinations. He was also ambulant with the aid of a walking frame. The medications were unchanged.

By June 2023, there was a further decline in cognition. This was accompanied by a deterioration in the patient’s ability to perform basic ADLs, with occasional urinary and bowel incontinence. Visual hallucinations persisted, but his sleep was unaffected. Unfortunately, the dyskinesia worsened, affecting his ability to consume food. This occurred without any known trigger or change in medication.

At this point, we suspected possible sensitivity to SSRIs. Hence, the escitalopram was stopped. Zolpidem, a sedative-hypnotic, was commenced for sleep, which was started at 5 mg nightly and subsequently increased to 10 mg. Teleconsultation after 2 weeks revealed improvements in agitation, hallucinations, and dyskinesia. He remained on zolpidem and donepezil only.

Most recently, in December 2023, the patient showed stable cognition and no BPSD. Sleep was manageable. He remained capable of self-care with minimal assistance. Dyskinesia was still present but mild and manageable, with no significant impact on his daily activities.

We continued the donepezil and added memantine 20 mg daily, an N-methyl-D-aspartate (NMDA) receptor antagonist, to better manage his cognitive impairment and BPSD. The zolpidem was continued. The carer expressed satisfaction with the patient’s progress, as he was significantly more manageable.

**Figure 1:**
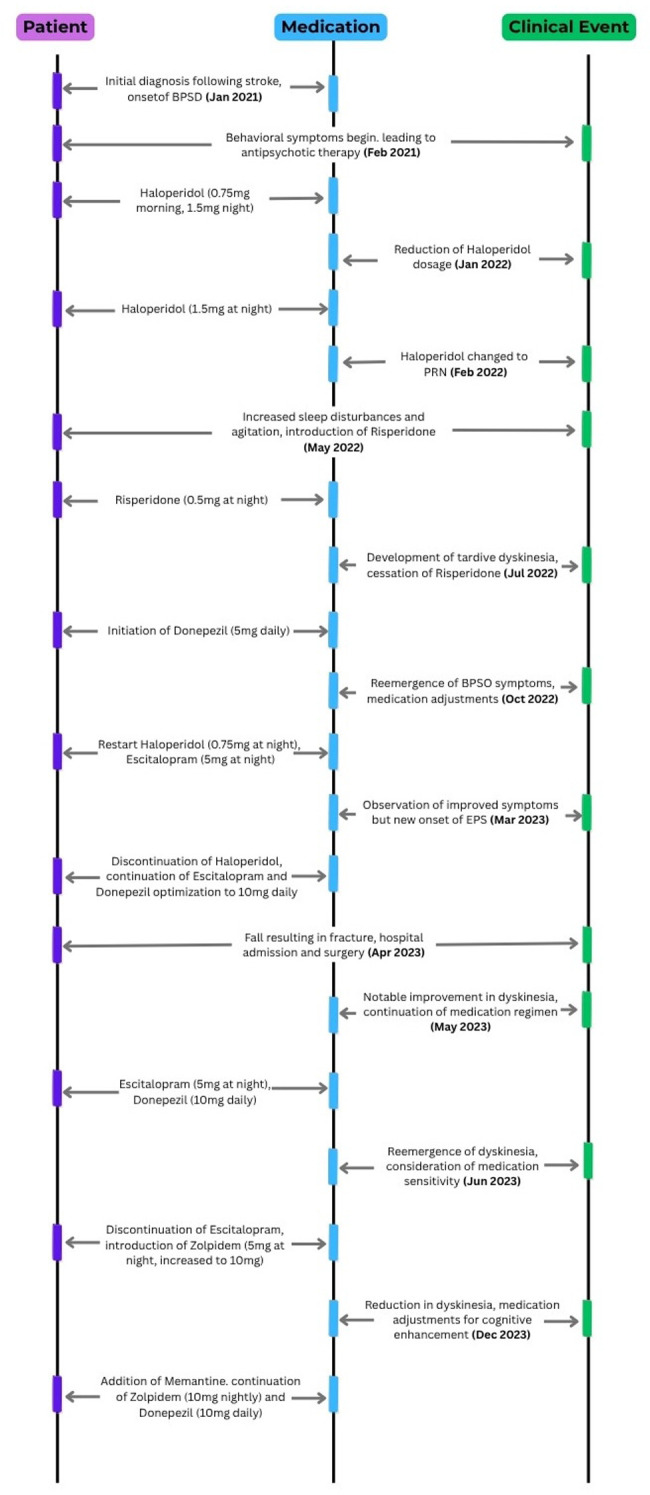
Event and medication.

The events and medication adjustment timeline are shown in Figure 1.

## Discussion

This case highlights the real-world complexity of managing BPSD in older adults requiring pharmacological intervention. TD is a recognized phenomenon; however, the fluctuating and recurrent pattern observed in this patient raises the possibility of multiple contributing factors.

Initial symptom control was achieved with haloperidol, followed by risperidone. However, after approximately 6 weeks of risperidone therapy, the patient developed TD, leading to its discontinuation. Although the dyskinesia initially resolved, it later recurred after haloperidol was reintroduced in combination with escitalopram, and subsequently persisted despite cessation of antipsychotic therapy. At the same time, the patient remained on escitalopram and donepezil. This case serves as a reminder that in older adults, the balance between symptom control and adverse effects is often difficult to achieve, particularly in the presence of multiple interacting medications and underlying neurodegenerative disease.

TD is a neurological disorder characterized by involuntary and repetitive movements that predominantly affect the face, jaw, and oral cavity. Lingual involvement is less common, accounting for up to 20% of cases. Medications associated with TD and EPS span multiple drug classes, with varying levels of risk, as summarized in Table 1 [5]. Typical antipsychotics remain the highest-risk agents, while other commonly prescribed medications, including atypical antipsychotics and certain non-psychiatric drugs, may also contribute to TD, particularly in older adults and with prolonged exposure.

**Table 1:** Medications Associated with TD and EPS

Risk category	Medication class	Examples	Key notes
High risk	Typical antipsychotics	Haloperidol	Strong D2 blockade; highest association with TD and EPS
	Dopamine antagonist antiemetics	Metoclopramide, prochlorperazine	Significant TD risk, especially with prolonged use (> 12 weeks)
Moderate risk	Atypical antipsychotics	Risperidone, olanzapine, aripiprazole	Lower risk than typical APDs but still associated; some agents reported to induce TD
	Antidepressants	SSRIs (fluoxetine, sertraline), TCAs (amitriptyline, clomipramine)	Risk higher in older adults and with long-term exposure
	MAO inhibitors	Selegiline, rasagiline, phenelzine	Associated with dyskinesia, especially with dopaminergic interaction
	Mood stabilizers	Lithium (especially with APDs)	Increased risk when combined with antipsychotics
Low but notable risk	Anticholinergics	Procyclidine, trihexyphenidyl	May worsen TD and cognitive impairment
	Anticonvulsants	Phenytoin, carbamazepine, lamotrigine	Rare but reported; possibly underdiagnosed
	Antihistamines	Hydroxyzine	Risk with prolonged use, especially in older adults
	Antiparkinsonian drugs	L-DOPA	Dyskinesia (LID), dose- and duration-related
Rare/context-dependent risk	Decongestants	Pseudoephedrine, phenylpropanolamine	May exacerbate movement disorders
	Antimalarials	Chloroquine, amodiaquine	Mechanism unclear; possible neurotransmitter disruption
	Anxiolytics	Benzodiazepines (withdrawal), barbiturates	Withdrawal-emergent dyskinesia
	Stimulants	Amphetamines, methamphetamine	Dopaminergic neurotoxicity; persistent dyskinesia possible

Medications associated with TD and EPS, categorized according to relative risk based on available literature. Risk stratification reflects general trends reported in the literature and may vary depending on patient factors such as age, comorbidities, duration of exposure, and polypharmacy. APDs: antipsychotic drugs; L-DOPA: L-3,4-dihydroxyphenylalanin; LID: L-DOPA-induced dyskinesia; MAO: monoamine oxidase; SSRIs: selective serotonin uptake inhibitors; TCAs: tricyclic antidepressants.

The fluctuating course observed in this case raises important considerations regarding the role of psychotropic combinations, delayed drug effects, and increased medication sensitivity in older adults. These factors likely act in combination rather than as isolated contributors.

First, the delayed effects of the typical antipsychotic haloperidol were considered. These drugs strongly bind to dopamine D2 receptors, leading to chronic dopamine blockade. The risks of EPS and TD are well known, especially in older adults and with long-term use. Therefore, high-potency typical antipsychotics should be used cautiously when alternatives exist. This aligns with evidence that dopamine receptor blockade remains a key mechanism in the development of TD [5, 6].

The criteria for TD diagnosis are set out in the Fifth Edition of the Diagnostic and Statistical Manual of Mental Disorders (DSM V), which notes that symptoms may continue or start even after cessation of the offending drug [5], reflecting the timeline observed in this patient. This is consistent with reports of delayed onset or persistence of TD even after drug discontinuation [7].

Secondly, the introduction of risperidone, an atypical antipsychotic, may have exacerbated the dopamine receptor hypersensitivity initiated by haloperidol. While atypical antipsychotics are generally associated with a lower risk of causing TD compared to haloperidol, they are not without risk, and sequential exposure may contribute to cumulative effects [8]. Similar patterns have been described in older adults, where sequential exposure to different antipsychotics may contribute to cumulative risk and complicate attribution [9].

Additionally, SSRIs, such as escitalopram, can interact with neuroleptics, resulting in a complex interplay between serotonergic and dopaminergic systems. This may relate to serotonergic–dopaminergic interactions and drug accumulation with prolonged use [10]. This accumulation heightens the risk of developing adverse neurological effects, especially in older adults with renal or hepatic impairments.

Acetylcholinesterase inhibitors (AChEi), such as donepezil, are not commonly associated with TD but may influence susceptibility through modulation of central cholinergic activity. In the presence of dopamine receptor blockade, this altered neurotransmitter balance may increase susceptibility to movement disorders in vulnerable individuals [11, 12].

**Figure 2:**
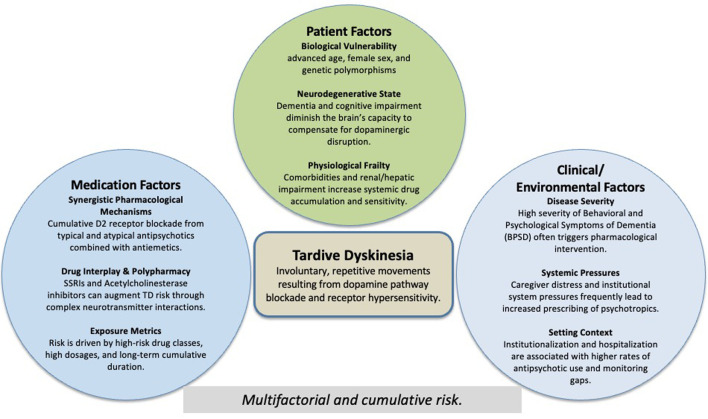
Multifactorial contributors to tardive dyskinesia in older adults with dementia. Conceptual model showing the multifactorial and cumulative contributors to tardive dyskinesia in older adults with dementia, including patient vulnerability, pharmacological exposure, and clinical/environmental factors.

The interplay of these contributing factors is summarized in Figure 2. In particular, patient-related vulnerability, including advanced age and underlying cognitive impairment, plays a key role in increasing susceptibility to TD. This increased vulnerability has been consistently reported in geriatric populations, particularly among those with neurodegenerative disease, with reported rates of EPS ranging from approximately 20% to 40% in individuals exposed to antipsychotic medications, especially typical agents. Although less common, TD remains an important long-term complication in this population [6, 13]. In our patient, the combination of vascular dementia and antipsychotic exposure likely reduced the capacity to compensate for dopaminergic disruption, while neurodegenerative processes may have further exacerbated receptor hypersensitivity [11].

Current guidelines emphasize non-pharmacological approaches as first-line management for BPSD, with pharmacological therapy reserved for severe or distressing symptoms, reflecting the limited efficacy and potential risks associated with these agents [14]. No pharmacological agent has demonstrated consistent efficacy or clear approval for this indication, and all carry significant adverse effects [12]. Despite this, clinicians are often compelled to prescribe these medications in the face of challenging behavioral symptoms, reflecting the realities of clinical practice.

The clinical challenge in this case extends beyond the occurrence of TD and reflects the complexity of selecting appropriate pharmacological therapy for BPSD in older adults. The selection of antipsychotic therapy in BPSD requires a careful balance between potential benefits and adverse effects. For example, conventional antipsychotics are associated with a higher risk of EPS, while atypical agents may carry increased cardiovascular and cerebrovascular risks. Therefore, treatment decisions should be individualized, taking into account patient-specific factors, symptom severity, and each agent’s risk profile.

In clinical practice, principles such as “start low and go slow,” careful dose titration, and regular early follow-up after initiation or dose adjustment are essential to minimize adverse effects in older adults [14]. Early recognition and management of EPS is crucial to the patient’s well-being and compliance with treatment. Close monitoring, particularly in the early weeks, is essential to detect adverse effects and guide timely adjustment. Teleconsultation, as implemented in our center, proved valuable for older adults with dementia, enabling regular monitoring of medication effects and side effects while reducing the need for frequent in-person visits.

Caution needs to be exercised when switching or combining antipsychotic medications, as the risks of EPS may be amplified. Recent guidelines and consensus statements continue to reinforce this cautious approach, although evidence to guide optimal pharmacological strategies remains limited. This highlights an important gap in the literature and the need for further research to support safer and more individualized management in this population.

Ultimately, this case emphasizes the need to balance the benefits of pharmacological treatment with the risk of side effects, while maintaining the patient’s quality of life.

### Conclusion

This case report illustrates the multifaceted challenges of managing dementia pharmacologically, particularly in addressing BPSD and the associated risk of TD. While existing guidelines provide a framework, the complex and heterogeneous nature of older adults with dementia demonstrates the limitations of a “one-size-fits-all” approach. Effective management requires a patient-centered strategy that balances symptom control with minimizing potentially debilitating side effects. This critical gap emphasizes the need for flexible therapeutic strategies that adapt to the complexities of comorbidities and polypharmacy. These challenges call for greater collaboration among clinicians, caregivers, and researchers to refine management strategies and enhance outcomes that address the realities of dementia care.

### Learning points

The management of BPSD in older adults is often complex and evolving, particularly when pharmacological treatment is required. In practice, balancing symptom control against the risk of adverse effects is rarely straightforward and may involve multiple adjustments and unintended consequences. This case highlights the importance of recognizing that medication-related complications such as TD may follow a fluctuating and unpredictable course, especially in the context of polypharmacy and underlying neurodegenerative disease. A cautious, individualized, and regularly reviewed approach to prescribing is therefore essential.

## Data Availability

The data supporting the findings of this study are available from the corresponding author upon reasonable request.
